# Complete Genome Sequence of *N*-Acylhomoserine Lactone-Producing *Pseudomonas* sp. Strain StFLB209, Isolated from Potato Phyllosphere

**DOI:** 10.1128/genomeA.01037-14

**Published:** 2014-10-16

**Authors:** Tomohiro Morohoshi, Taro Kato, Nobutaka Someya, Tsukasa Ikeda

**Affiliations:** a Department of Material and Environmental Chemistry, Graduate School of Engineering, Utsunomiya University, Utsunomiya, Tochigi, Japan; b Hokkaido Agricultural Research Center, National Agriculture and Food Research Organization, Memuro-cho, Kasai-gun, Hokkaido, Japan

## Abstract

*Pseudomonas* sp. strain StFLB209 is isolated from the potato leaf and produces *N*-acylhomoserine lactone quorum-sensing signal compounds. Here, we present the 6,332,373-bp complete genome sequence of StFLB209, with a G+C content of 60.7%, which carries 5,598 protein-coding genes, 6 rRNA operons, and 69 tRNA genes.

## GENOME ANNOUNCEMENT

In many Gram-negative bacteria, *N*-acylhomoserine lactones (AHLs) have been identified as signal compounds involved in quorum sensing (1). Several *Pseudomonas* species produce AHLs that regulate the expression of many genes and are responsible for pathogenicity, biofilm development, rhizosphere colonization, production of antibiotics, and other processes (2). Fluorescent pseudomonads are widely known to be beneficial to plants as biological control agents of plant diseases or as plant growth-promoting agents (3). In previous work, we reported the community structure of AHL-producing fluorescent pseudomonads in the potato rhizosphere and phyllosphere (3, 4). Based on the results of the phylogenetic analysis, 16S rRNA sequence of the most dominant phyllosphere species showed a certain degree of similarity to the opportunistic plant pathogen *Pseudomonas cichorii* (4). These dominant strains caused leaf necrosis and putrefaction of tuber slices as well as *P. cichorii* (4). The complete genome sequence of *P. cichorii* JBC1 has been deposited in DDBJ/ENA/GenBank databases (accession number CP007039) in the previous study (5). Here, we report the complete genome sequence of one of the dominant strains in the potato phyllosphere, *Pseudomonas* sp. StFLB209.

Single- and paired-end whole-genome shotgun sequencing of StFLB209 was performed using Roche Genome Sequencer FLX Titanium pyrosequencing technology (6) by Eurofins Genomics (Tokyo, Japan). We produced 1,026,084 reads with an average read length of 210 bases. The total number of sequenced bases is 216,076,150, representing a sequencing depth of 34×. Using the Celera Assembler version 5.3, these reads were assembled into one large scaffold including 31 large contigs (>1,000 bp). Gap closure was attempted using gap-spanning clones and PCR products. Prediction of putative coding sequences and gene annotation were done using the Microbial Genome Annotation Pipeline (http://www.migap.org/). Briefly, protein-coding sequences (CDSs) were predicted by the combined use of MetaGeneAnnotator (7), RNAmmer (8), tRNAScan (9), and BLAST (10). The complete genomic information of *Pseudomonas* sp. StFLB209 is contained on a single circular chromosome of 6,332,373 bp with an average G+C content of 60.7%. The genome contains 5,598 protein-coding genes, 6 rRNA operons, and 69 tRNA genes. We searched for homologs of the reported AHL synthase gene in the complete genome sequence of StFLB209. One predicted coding sequence (PSCI_0294), which encoded 197 amino acids, showed 85.8% identity with PpuI from *Pseudomonas putida* IsoF (11). On the other hand, any AHL synthase gene homologs were not identified in the complete genome sequence of *P. cichorii* JBC1. Further studies on AHL-mediated quorum-sensing-regulated gene expression are needed to understand the mechanisms underlying the pathogenicity of StFLB209.

### Nucleotide sequence accession number.

The complete genome sequence of *Pseudomonas* sp. strain StFLB209 has been deposited in the DDBJ/ENA/GenBank databases under accession number AP014637.

## References

[1] Waters CM Bassler BL. 2005. Quorum sensing: cell-to-cell communication in bacteria. Annu. Rev. Cell Dev. Biol. 21:319–346. 10.1146/annurev.cellbio.21.012704.131001 16212498

[2] Venturi V. 2006. Regulation of quorum sensing in *Pseudomonas*. FEMS Microbiol. Rev. 30:274–291. 10.1111/j.1574-6976.2005.00012.x 16472307

[3] Someya N Morohoshi T Ikeda T Tsuchiya K Ikeda S. 2012. Genetic diversity and ecological evaluation of fluorescent pseudomonads isolated from the leaves and roots of potato plants. Microbes Environ. 27:122–126. 10.1264/jsme2.ME11237 22791043 PMC4036014

[4] Someya N Morohoshi T Okano N Otsu E Usuki K Sayama M Sekiguchi H Ikeda T Ishida S. 2009. Distribution of *N*-acylhomoserine lactone-producing fluorescent pseudomonads in the phyllosphere and rhizosphere of potato (*Solanum tuberosum* L.). Microbes Environ. 24:305–314. 10.1264/jsme2.ME09155 21566390

[5] Ramkumar G Lee S Weon HY Kim BY Lee Y. 4 7 2014. First report on the whole genome sequence of *Pseudomonas cichorii* strain JBC1 and comparison with other *Pseudomonas* species. Plant Pathol. 10.1111/ppa.12259

[6] Margulies M Egholm M Altman WE Attiya S Bader JS Bemben LA Berka J Braverman MS Chen YJ Chen Z Dewell SB Du L Fierro JM Gomes XV Godwin BC He W Helgesen S Ho CH Irzyk GP Jando SC Alenquer ML Jarvie TP Jirage KB Kim JB Knight JR Lanza JR Leamon JH Lefkowitz SM Lei M Li J Lohman KL Lu H Makhijani VB McDade KE McKenna MP Myers EW Nickerson E Nobile JR Plant R Puc BP Ronan MT Roth GT Sarkis GJ Simons JF Simpson JW Srinivasan M Tartaro KR Tomasz A Vogt KA Volkmer GA Wang SH Wang Y Weiner MP Yu P Begley RF Rothberg JM. 2005. Genome sequencing in microfabricated high-density picolitre reactors. Nature 437:376–380. 10.1038/nature03959 16056220 PMC1464427

[7] Noguchi H Taniguchi T Itoh T. 2008. MetaGeneAnnotator: detecting species-specific patterns of ribosomal binding site for precise gene prediction in anonymous prokaryotic and phage genomes. DNA Res. 15:387–396. 10.1093/dnares/dsn027 18940874 PMC2608843

[8] Lagesen K Hallin P Rødland EA Staerfeldt HH Rognes T Ussery DW. 2007. RNAmmer: consistent and rapid annotation of ribosomal RNA genes. Nucleic Acids Res. 35:3100–3108. 10.1093/nar/gkm160 17452365 PMC1888812

[9] Lowe TM Eddy SR. 1997. tRNAscan-SE: a program for improved detection of transfer RNA genes in genomic sequence. Nucleic Acids Res. 25:955–964. 10.1093/nar/25.5.0955 9023104 PMC146525

[10] Altschul SF Gish W Miller W Myers EW Lipman DJ. 1990. Basic local alignment search tool. J. Mol. Biol. 215:403–410. 10.1016/S0022-2836(05)80360-2 2231712

[11] Steidle A Allesen-Holm M Riedel K Berg G Givskov M Molin S Eberl L. 2002. Identification and characterization of an *N*-acylhomoserine lactone-dependent quorum-sensing system in *Pseudomonas putida* strain IsoF. Appl. Environ. Microbiol. 68:6371–6382. 10.1128/AEM.68.12.6371-6382.2002 12450862 PMC134430

